# Neuronal nicotinic acetylcholine receptors: neuroplastic changes underlying alcohol and nicotine addictions

**DOI:** 10.3389/fnmol.2012.00083

**Published:** 2012-08-03

**Authors:** Allison A. Feduccia, Susmita Chatterjee, Selena E. Bartlett

**Affiliations:** ^1^Ernest Gallo Clinic and Research Center, Preclinical DevelopmentEmeryville, CA, USA; ^2^Circuit Therapeutics, MountainviewCA, USA; ^3^Translational Research Institute at Institute of Health and Biomedical Innovation, Queensland University of TechnologyBrisbane, QLD, Australia

**Keywords:** addiction, behavioral sensitization, cholinergic, ethanol, neuroplasticity, nicotine, nicotinic acetylcholine receptors, synaptic transmission

## Abstract

Addictive drugs can activate systems involved in normal reward-related learning, creating long-lasting memories of the drug's reinforcing effects and the environmental cues surrounding the experience. These memories significantly contribute to the maintenance of compulsive drug use as well as cue-induced relapse which can occur even after long periods of abstinence. Synaptic plasticity is thought to be a prominent molecular mechanism underlying drug-induced learning and memories. Ethanol and nicotine are both widely abused drugs that share a common molecular target in the brain, the neuronal nicotinic acetylcholine receptors (nAChRs). The nAChRs are ligand-gated ion channels that are vastly distributed throughout the brain and play a key role in synaptic neurotransmission. In this review, we will delineate the role of nAChRs in the development of ethanol and nicotine addiction. We will characterize both ethanol and nicotine's effects on nAChR-mediated synaptic transmission and plasticity in several key brain areas that are important for addiction. Finally, we will discuss some of the behavioral outcomes of drug-induced synaptic plasticity in animal models. An understanding of the molecular and cellular changes that occur following administration of ethanol and nicotine will lead to better therapeutic strategies.

## Introduction

**Figure 1:**
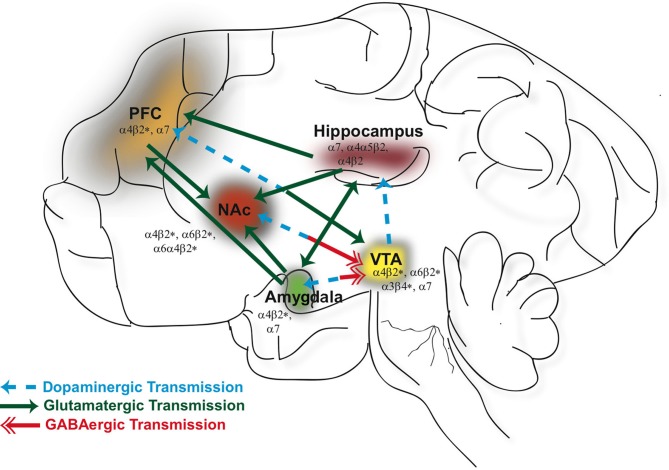
**Neuronal nicotinic acetylcholine receptors (nAChRs) are widely distributed in different brain regions that include the ventral tegmental area (VTA), nucleus accumbens (NAc), hippocampus, prefrontal cortex (PFC), and amygdala.** Activation of nAChRs in these brain areas significantly contribute to the rewarding effects of ethanol and nicotine and play a role in modulating synaptic plasticity. GABAergic (red), glutamatergic (green), and dopaminergic (blue) connections between these structures constitute a major neural circuitry underlying addictive disorders.

For the past several decades researchers have put forth concerted efforts to investigate the effects of drugs of abuse on brain function with the ultimate goal of developing medications useful in terminating drug use and preventing relapse. The progression from initial drug use to drug dependence involves complex, multifaceted neural adaptations that encompass molecular changes at the cellular level within several different brain circuits. From studies involving human subjects and animal models, drugs of abuse are known to act on the cortico-limbic network (see Figure 1) that governs reward (mesolimbic dopaminergic pathway), learning and memory (hippocampus), emotion (amygdala), and executive functions (prefrontal cortex). Our understanding, at this point, remains limited regarding how drug-related experiences are encoded in the brain and their relationship to synaptic plasticity and the development of addictions.

It is now generally accepted that addiction is a type of learning, i.e., learned associations between the rewarding effects of drugs and environmental cues that predict drug availability, which significantly contributes to compulsive drug use and the propensity to relapse even after long periods of abstinence. As subjects make the transition from initiation to habitual drug use, neuro-adaptations in areas such as the hippocampus and amygdala are thought to underlie drug-associated learning and memories. A single episode of drug use itself can influence synaptic transmission and repeated or prolonged drug use can cause long-lasting alterations in synaptic strengths—defined as synaptic plasticity, reflected through molecular changes as well as persistent modification of neurotransmitter release. These drug-induced changes in the brain are a critical component in the development of dependence and are thought to drive compulsive intake and relapse. There are many factors that play into addictive processes and understanding the neurobiological underpinnings of these events will enlighten possible therapeutic targets.

The most well-known form of synaptic plasticity, NMDAR-dependent, was first discovered in 1973 in the hippocampi of anesthetized rats (Bliss and Lomo, 1973) and has since been extensively characterized from electrophysiological recordings of *in vitro* hippocampal slice preparations. Long-term potentiation (LTP) is defined as an increase in the post-synaptic response resulting from a cascade of events which is initiated by an influx of Ca^2+^ ions through voltage-gated pre-synaptic ion channels. The NMDAR-dependent form of LTP requires postsynaptic depolarization during NMDAR activation, which will allow for an influx of Ca^2+^ ions through the channel within the dendritic spine. This increase of Ca^2+^ will commence a series of intracellular signaling, activating a number of protein kinases and consequently lead to the insertion of AMPA receptors into the plasma membrane. In contrast, long term depression (LTD) is attributed to a weak activation of NMDARs, minimal Ca^2+^ influx, and a reduction in post-synaptic AMPA surface receptor density via dynamin- and clathrin-dependent endocytosis (Malenka and Bear, 2004). Much of the NMDAR-dependent plasticity has focused on mechanisms responsible for the initial increase in synaptic strength, however the long-lasting biological effects likely require new protein synthesis and gene transcription (Lynch, 2004). There are quantifiable alterations in the morphology of dendrites and dendritic spines that accompany LTP (Andersen and Soleng, 1998; Yuste and Bonhoeffer, 2001; Matsuzaki et al., 2004) and together these long-lasting changes have been implicated as an essential mechanism and molecular basis for learning and memory. Additionally, there are other modulators of plasticity, such as metabotropic glutamate receptors and endocannabinoids, that have been discovered and reviewed extensively elsewhere [see review, Kauer and Malenka (2007)].

Alcohol and tobacco addiction are among the highest causes of preventable death worldwide (Mokdad et al., 2004) and the comorbidity of these two substance abuse disorders is striking (DiFranza and Guerrera, 1990; Batel et al., 1995; Falk et al., 2006). While the easy availability and low social stigma of alcohol and cigarettes provides an explanation of their high prevalence of dual dependence, strong neurobiological evidence suggests a common link between these two substances (de Fiebre et al., 1990; Smith et al., 1999; Gould et al., 2001; Marubio et al., 2003; Tizabi et al., 2007). Neuronal nicotinic acetylcholine receptors (nAChRs) are widely expressed throughout the brain (Gotti et al., 2007) and are suggested to be the common biological target of nicotine and ethanol (Tapper et al., 2004; Funk et al., 2006; Steensland et al., 2007; Bito-Onon et al., 2011). nAChRs are pentameric ligand-gated ion channels, consisting of various heteromeric or homomeric combinations of α (α_2_–α_10_) and β (β_2_–β_4_) subunits (Albuquerque et al., 2009; Gotti et al., 2009). Most neuronal nAChRs are heteromeric receptors with just two binding sites, but some subunits, such as the α7, form functional homomeric receptors with five binding sites (Changeux, 2009; Gotti et al., 2009). The most abundant of nAChR subtypes in the brain are the α4β2^*^ (^*^indicates the possibility of other subunits) followed by the α7; correspondingly, the mRNA of these subtypes are found throughout the entire brain. The vast regional distribution and location of nAChRs are thoroughly reported in the following reviews (Gotti and Clementi, 2004; Gotti et al., 2007).

Binding of endogenous acetylcholine (ACh) or nicotine induces a conformational change of the receptor allowing for an influx of cations (Ca2+, Na+, or K+ depending on nAChR subtype) through the central channel for a few milliseconds, followed by a non-conducting closed receptor state (Giniatullin et al., 2005). In contrast, ethanol is not a direct agonist at nAChRs but can potentiate the response of these receptors to Ach (Aistrup et al., 1999; Cardoso et al., 1999; Zuo et al., 2002). The pharmacological properties of the nAChRs to agonists such as ACh, nicotine or ethanol is highly dependent on its subunit composition and location of the receptor (Yu et al., 1996; Pidoplichko et al., 1997; Cardoso et al., 1999; Wooltorton et al., 2003).

Nicotinic receptors are localized both pre- and post-synaptically where agonist-induced cation influx results in membrane depolarization and/or CA^2+^-dependent signaling cascades, thereby regulating neuronal excitability and neurotransmitter release (Albuquerque et al., 1995). nAChRs play a significant role in modulating glutamatergic, GABAergic, and dopaminergic neurotransmission in the mesolimbic pathway (Blomqvist et al., 1992; Caille et al., 2009; Mao et al., 2011), thus contributing to the rewarding effects of drugs of abuse and synaptic plasticity in this system as well as in other brain regions such as the hippocampus and amygdala (Ericson et al., 2009; Hendrickson et al., 2010; Reperant et al., 2010). Moreover upon exposure to nicotine and ethanol, nicotinic receptors can undergo changes in expression (stoichiometries or receptor number) and function which may underlie aspects of physical dependence and withdrawal symptoms (Nashmi et al., 2007). Since the majority of alcoholics are also smokers, determining the coincident molecular underpinnings in the development of their dependence may be useful in treating these addictions.

This review will attempt to consolidate the available information regarding nicotinic receptor-mediated synaptic plasticity and integrate these enduring neural adaptations with current models of addictive disorders. We will look at a myriad of addictive processes, the underlying neural circuits and how these pathways converge for addictive behaviors to emerge. The complexity of addictive disorders suggests there are a considerable number of possible targets for intervention that could potentially reverse drug-induced neural adaptations. We will aim to focus this paper towards nicotinic receptor-mediated neuroplasticity, with an emphasis on nicotine and ethanol.

## Nicotine and ethanol: nAChR-mediated neurotransmission and plasticity

In addition to alterations in glutamatergic signaling, there is ample evidence showing other neurotransmitter systems, such as the cholinergic and dopaminergic, play a vital role in modulating the induction, duration, and magnitude of synaptic plasticity (Otani, 2003; Drever et al., 2011). ACh acts on a variety of different pre- and post-synaptic receptors throughout the brain resulting in profoundly different outcomes depending on receptor location and subunit composition (Alkondon and Albuquerque, 2004). Thus far, most studies have implicated the involvement of G-protein coupled muscarinic receptors in mediating these synaptic changes; however, more recently nAChRs have come under investigation to understand their part in these processes. In this section, the role of nAChRs in the modulation of neurotransmission and drug-induced plasticity will be discussed for the brain loci that have been implicated to be important for the development of nicotine and ethanol addiction.

### Midbrain: reward pathway

The mesolimbic dopaminergic system, encompassing DA neurons originating from the ventral tegmental area (VTA) that project to the nucleus accumbens (NAc) and PFC, has long been recognized as an important pathway mediating behavioral responses to natural rewards as well as drugs of abuse. Essentially, all drugs of abuse enhance extracellular DA in the NAc (Di Chiara and Imperato, 1988) and blocking dopaminergic transmission will attenuate the reinforcing properties of the drug (Corrigall et al., 1992). Furthermore, this DA signal provides convergent information to the system regarding reward expectation and environmental cues related to drug intake (Di Chiara, 1999; Berke and Hyman, 2000). It is not surprising, therefore that drug-induced neural adaptations have been discovered in this circuit and presumably contribute to addiction.

#### Ventral tegmental area

**Figure 2:**
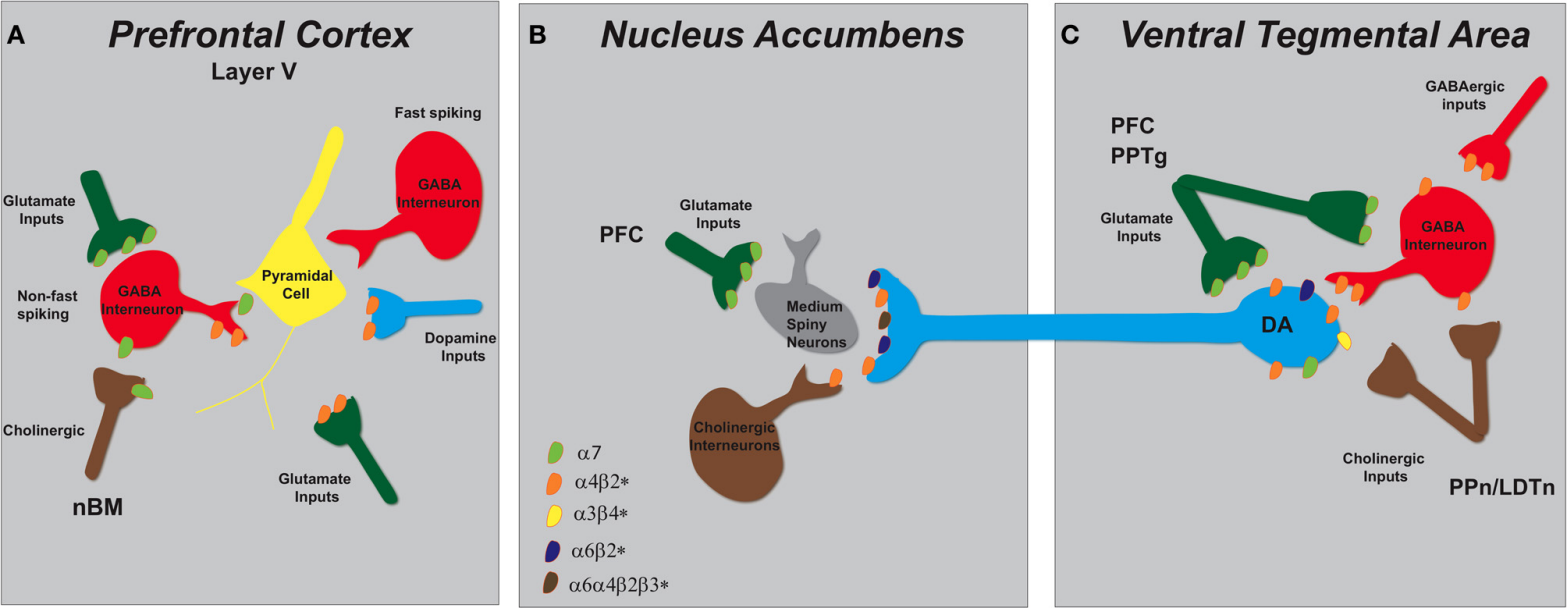
**Schematic representation of nAChR subtypes and circuit function in the mesolimbic dopaminergic system. (A)** Pyramidal cells in layer V of the PFC lack nAChRs but their activity is modulated by excitatory and inhibitory neurons that do express them. There are two types of GABAergic interneurons, fast spiking and non-fast spiking, with only the latter bearing nAChRs (α7 and α4β2^*^). Distinct populations of glutamatergic inputs express either α7 or α4β2^*^ nAChRs while DA terminals projecting from the VTA contain α4β2^*^ nAChRs. Cholinergic inputs into the PFC arise from the nucleus basalis of Meynert (nBM). **(B)** In the NAc, nAChRs (α4β2^*^, α6β2^*^, and α6α4β2^*^) expressed on DAergic terminals from the VTA mediate DA release based on the neuronal activity firing rate. A small population of tonically active cholinergic interneurons (~2%) is synchronized with DA cell firing. Glutamatergic inputs from the PFC endow α7 nAChRs. **(C)** The VTA receives cholinergic innervation from the pedunculopontine (PPn) and laterodorsal tegmental nuclei (LDTn). In addition to the nAChRs localized on DA cell bodies, DAergic cell firing is modulated by α4β2^*^ (and possibly α7) nAChRs expressed on GABAergic interneurons and excitatory glutamatergic afferents from the PFC and the PPn.

The VTA is modulated by excitatory glutamatergic inputs arising from the PFC, bed nucleus of the stria terminalis, amygdala, pontomesencephalic tegmental nuclei (Mao and McGehee, 2010), and by a large population of inhibitory GABAergic interneurons (Johnson and North, 1992; Theile et al., 2008) and afferents that arise from heterogeneous sources (Geisler and Zahm, 2005). There are several different nAChR subunits expressed in the VTA, some of which are the α3, α4, α5, α6, α7, β2, and β3 subtypes (Azam et al., 2002; Perry et al., 2002; Yang et al., 2009). The α4 and β2 mRNAs are expressed in nearly all DAergic and GABAergic VTA neurons, while α7 mRNA is distributed in only 40% of these neurons (Nashmi and Lester, 2006). The α7^*^ nAChRs are most densely localized on pre-synaptic glutamatergic, but not cholinergic, terminals in the VTA (see Figure 2) (Klink et al., 2001; Jones and Wonnacott, 2004).

Both ethanol and nicotine stimulate dopamine release in the accumbens by modulating the activity of VTA neurons via nAChRs (Champtiaux et al., 2003). In the presence of physiologically relevant doses of nicotine (100–500 nM) (Nguyen et al., 2003; Parker et al., 2004), nAChRs are briefly activated followed by rapid desensitization, which entails a reversible inactivation of response. Therefore, nicotine's mechanism of action and downstream consequences are attributed to both receptor activation and desensitization (Mansvelder et al., 2002). The propensity of these receptors to desensitize depends largely on the subunit composition of the nAChR assembly. α7 nAChRs have a much lower affinity for nicotine compared to β2-containing nAChRs and are less susceptible to desensitization in the presence of relevant smoking-related concentrations (Mansvelder and McGehee, 2002; Quick and Lester, 2002; Wooltorton et al., 2003).

The initial few minutes of nicotine exposure will enhance DA release in the NAc by activating α4β2^*^ nAChRs on dopamine neuronal soma (Pidoplichko et al., 1997; Mansvelder and McGehee, 2000); however, β2^*^-containing receptors rapidly desensitize. Simultaneously, nicotine acts on α4β2^*^ nAChRs located on GABAergic neurons to induce a transient inhibition of DA activity. These nicotinic receptors will also subsequently desensitize in the presence of nicotine with a net result being a reduction in the inhibitory control of GABA on dopaminergic transmission (Mansvelder and McGehee, 2002). On the same time scale, nicotine binds to α7 nAChRs present on glutamatergic terminals in the VTA, whose activation increases glutamate release onto NMDA-type glutamate receptors located on dopaminergic cell bodies and increases the frequency of spontaneous excitatory postsynaptic currents (sEPSCs) (Mansvelder and McGehee, 2000; Schilstrom et al., 2000; Marchi et al., 2002; Pidoplichko et al., 2004). Hence, the cumulative result of nicotine acting on nAChRs in the VTA is enhanced excitatory input onto DA neurons which triggers reward-related high frequency burst firing resulting in increased accumbal DA outflow (Corrigall et al., 1994; Nisell et al., 1994; Schilstrom et al., 2000; Pidoplichko et al., 2004).

Several labs have investigated the specific subunit compositions of the nAChRs that may be critical in the development of nicotine dependence using cell-based heterologous expression systems, transgenic mouse lines, and pharmacological manipulations using nAChR ligands in animal behavior models (Tapper et al., 2004; Steensland et al., 2007; Chatterjee and Bartlett, 2010; Cahir et al., 2011). The α4β2^*^ and α6β2^*^ nAChR subtypes play an essential role in mediating the rewarding effects of nicotine (Dani and De Biasi, 2001; Nashmi et al., 2007), demonstrated by a lack of nicotine-elicited DA release in β2 and α4 knockout mice and a decrease in nicotine self-administration by mice lacking the α4, α6, or β2 subunits compared to wild-types (Picciotto et al., 1998; Marubio et al., 2003; Pons et al., 2008). By contrast, the role α7 nAChRs play in the reinforcing properties of nicotine is less clear. Deletion of the α7 did not affect nicotine conditioned place preference (Walters et al., 2006) or self-administration (Pons et al., 2008); however, high doses of the α7 nAChR antagonist methyllycaconitine attenuated nicotine self-administration (Markou and Paterson, 2001) and reduced the rewarding effects of nicotine when infused directly into the VTA (Laviolette and van der Kooy, 2003).

The effects of ethanol in the VTA are more complex than the effects of nicotine. Ethanol, unlike nicotine, is not a direct agonist at nAChRs (Cardoso et al., 1999; Zuo et al., 2002) but can modulate DA release by influencing the function of nAChRs in both the VTA and NAc (Ericson et al., 1998, 2003; Larsson et al., 2005). While local perfusion of ethanol into the VTA does not increase DA release in the accumbens, infusion of ethanol into the accumbens does elevate extracellular DA to a similar degree as systemic administration (Ericson et al., 2003; Tuomainen et al., 2003). However, ethanol infused into both regions simultaneously resulted in higher DA levels than when injected into the NAc alone (Lof et al., 2007). Furthermore, perfusion of mecamylamine (MEC, non-selective nAChR antagonist) into the VTA, but not into the NAc, blocks DA release stimulated by systemic administration of ethanol. It has been postulated that ethanol's actions in the NAc may facilitate the release of endogenous ACh in the VTA, leading to activation of nAChRs and consequently elevating accumbal DA release (Larsson et al., 2005). In support, voluntary ethanol intake in rats has been shown to cause a significant increase in ACh levels in the VTA which is time-locked with the DA increase in the NAc (Larsson et al., 2005). It should be mentioned here that in addition to nAChRs, ethanol is known to modulate DA neurotransmission through actions at other molecular targets [for review (Vengeliene et al., 2008)]. For example, ethanol (10–80 mM) acting on pre-synaptic D_1_ receptors can increase excitatory glutamate transmission in the VTA and enhance DA release (Xiao et al., 2009).

While, it is relatively clear that α4β2^*^ and α7 nAChRs play a major role for nicotine mediated dopamine effects in the VTA, it has been more difficult to determine the specific nAChR compositions important for ethanol. Several subunit-specific antagonists have been administered to mice both systemically and by direct infusion into the brain. Ethanol-induced DA release in the NAc and locomotor activity were blocked by systemic injections of MEC but not by methyllycaconitine citrate (MLA, α7 antagonist) or dihydro-β-erythroidine (DHβE, broad-spectrum β2^*^-antagonist), suggesting ethanol's stimulatory effects may not be mediated by β2^*^-containing or α7 nAChRs (Larsson et al., 2002). Another study further defined which subunits were involved in ethanol's effects on the DAergic system by administering various α-conotoxins into the VTA and measuring DA outflow in the NAc following an ethanol challenge. Results showed that α-conotoxin MII, selective for α3β2^*^ and/or β3^*^ and/or α6^*^ subunits, significantly reduced ethanol-stimulated DA release in the NAc, while the selective α6^*^ antagonist, α-conotoxin PIA-analog, showed no effect (Larsson and Engel, 2004; Jerlhag et al., 2006). Taken together, this evidence suggests ethanol's actions in the VTA are mediated by α3β2^*^ and/or β3^*^, rather than α4β2^*^, α7, or α6^*^ nAChRs; however, it remains unclear if these effects are due to direct or indirect interactions of ethanol with nAChRs.

***nAChR-mediated synaptic plasticity.*** In addition to augmenting transmitter release, drugs of abuse can induce long-lasting plasticity within midbrain DA centers following both acute and chronic drug administration (Gao et al., 2010). In the VTA, drug-evoked NMDA-mediated plasticity may occur in a similar fashion to that seen in the hippocampus and requires activation of pre-synaptic voltage-gated ion channels. Indeed, several drugs of abuse can elicit LTP in VTA excitatory synapses and increase AMPA receptors without changing the number or function of NMDA receptors (Saal et al., 2003; Jin et al., 2011). Activation of nAChRs by nicotine likely influence the persistent potentiation of these excitatory synapses (Jin et al., 2011), however at this time it is unclear if and how nAChRs play a role in ethanol-mediated NMDAR-dependent plasticity.

There is experimental evidence illustrating nicotine-induced plasticity (Table 1) in VTA slice preparations where application of nicotine (500 nM) was shown to increase the AMPA/NMDA receptor ratio by increasing pre-synaptic release of glutamate. This effect is mediated by α7 nAChRs since pre-incubation with the α7 antagonist (MLA), but not the β2^*^-antagonist (DHβE), abolished the nicotine-induced LTP. In support, α7 knockout mice lack this response to nicotine (Jin et al., 2011). Furthermore, the nicotine-induced enhancement of excitatory currents lasts after prolonged exposure suggesting a lack of α7 desensitization (Pidoplichko et al., 2004) and persists even after nicotine is removed from the bath, thereby indicating LTP (Mansvelder and McGehee, 2000; Dani, 2001; Mansvelder et al., 2002).

**Table 1:** **nAChRs modulate synaptic transmission in the mesolimbic system**.

**Location**	**nAChR subtype**	**Agonist**	**Outcome**	**Mechanism**	**References**
Ventral tegmental area	α7 on presynaptic glutamatergic neurons	Nicotine	↑ Dopamine (DA) release in the nucleus accumbens Promotes long-term potentiation (LTP)	↑ glutamate release onto NMDARs located on DAergic cell bodies ↑ frequency of spontaneous postsynaptic currents (sEPSCs)	Mansvelder and McGehee, 2000; Schilstrom et al., 2000; Pidoplichko et al., 2004
Nucleus accumbens		Nicotine	↑ dendritic length and branches		Brown and Kolb, 2001; McDonald et al., 2007
Prefrontal cortex	Activation of nAChRs on soma of GABAergic interneurons	Nicotine	↑ threshold for induction of spike-timing-dependent plasticity	↑ GABAergic inputs to PFC layer 5 pyramidal neurons ↑ inhibitory postsynaptic currents (IPSCs) Reduces post-synaptic Ca^2+^ signals	Couey et al., 2007
	Activation of nAChRs on glutamatergic terminals	Nicotine	↑ threshold for induction of spike-timing-dependent plasticity	↑ glutamate release onto fast spiking interneurons ↑ GABAergic inputs to PFC layer 5 pyramidal neurons ↑ IPSCs Reduces post-synaptic Ca^2+^ signals	Couey et al., 2007
		Nicotine	↑ dendritic length and branches		Brown and Kolb, 2001

#### Striatum

The striatum is a heterogeneous structure, with distinct anatomical and functional subterritories that can be broadly classified into the dorsal and ventral striatum. The majority (>90%) of the neurons in the striatum are GABAergic medium spiny neurons (MSN) with most (~80%) of the synapses being asymmetric glutamatergic inputs (Wilson, 2007; Tepper et al., 2008). In addition, there are at least three other types of interneurons, including a small population of cholinergic interneurons (~2%) that provide a rather extensive arborization in this region to modulate DA release probability (Zhou et al., 2002; Pakhotin and Bracci, 2007). The dopaminergic neurons arising from the VTA project to the ventral striatum (NAc and part of the olfactory tubercle), while the dorsal striatum (caudate-putamen) receives dopaminergic inputs primarily from the substantia niagra pars compacta. The ventral striatum is highly involved in the reinforcing effects of drugs of abuse and receives extensive excitatory afferents from the PFC, amygdala and hippocampus (Carelli, 2002; Volkow et al., 2006). On the other hand, efferent projections from the motor cortex to the dorsal striatum allows this subregion to gate sensorimotor function and have been implicated in the advanced stages of habitual drug seeking (Fasano and Brambilla, 2002; Gerdeman et al., 2003; Philibin et al., 2011).

There is a limited number of subunits (α4, α5, α6, β2, and β3) densely localized on pre-synaptic DAergic axon terminals in the striatum (Grady et al., 2007), where β2-containing nAChRs can directly influence local DA release based on the neuronal activity firing rate (see Figure 2) (Zoli et al., 2002; Rice and Cragg, 2004). In the proposed model, ACh released from tonically active striatal cholinergic interneurons normally acts on these receptors to gate the probability of DA release and enhance the contrast between tonic and phasic firing patterns. In a manner similar to those in the VTA, these β2-nAChRs will rapidly desensitize following agonist application. Initial nicotine administration causes a reduction of dopamine release from tonically active neurons; however, a reward-related burst of action potentials will result in even greater transmitter release due to loss of the normal modulatory control of endogenous Ach (Zhou et al., 2001; Rice and Cragg, 2004; Salminen et al., 2004; Zhang and Sulzer, 2004).

Besides anatomical and connectivity differences, there are clear distinctions in DA signaling between the ventral and dorsal striatum which govern their specific brain functions and behavioral output. For example, initial drug use will favor DA release in the NAc shell rather than the dorsolateral striatum (Pontieri et al., 1996; Di Chiara, 2002). Furthermore, these two regions respond differently to stimulus trains that mimic action potentials, with tonic and phasic firing eliciting greater DA release from the dorsolateral striatum and NAc shell, respectively (Zhang et al., 2009). Recently, it was demonstrated that activity-dependent DA transmission in the ventral striatum is mainly controlled by α6^*^-containing nAChRs (α6α4β2β3 and α6β2^*^) but in the dorsal striatum non-α6 nAChRs (α4β2 and α4α5β2) are the key players (Exley et al., 2007, 2008, 2011). In summary, DA release in the striatum is modulated by different nAChRs on pre-synaptic DAergic terminals in a frequency-dependent and region-specific manner.

***nAChR-mediated synaptic plasticity.*** In the NAc, synapses can express both LTP and LTD (Kombian and Malenka, 1994) but mainly NMDAR-dependent LTD has been demonstrated following administration of psychostimulants, such as cocaine and amphetamine, which involves the endocytosis of AMPARs. Although in some situations ethanol has been shown to inhibit NMDAR activity and associated plasticity (Blitzer et al., 1990), activation of DA D1 receptors will diminish ethanol's inhibition of NMDARs thus promoting reinforcement and plasticity in the NAc (Maldve et al., 2002). Ethanol treatment (50 mM) has been shown to cause a short-term depression of the striatal output and this effect is sensitive to nAChR antagonists, MEC (10 μM) and MLA (40 nM), which block the depression of synaptic output (Adermark et al., 2011).

Neuronal morphological changes, including increases in spine density or dendritic length, have been reported following environmental enrichment or drug administration (Johansson and Belichenko, 2002; Leggio et al., 2005) and are thought to reflect structural reorganization of neural circuits manifested by molecular events discussed thus far in this review. In the NAc, long-lasting structural plasticity has been demonstrated in MSN following nicotine administration; however, these changes may be dependent on treatment schedule and cohort age. One study found an increase in dendritic length and spine density in the NAc and PFC of adult rats after intermittent subcutaneous injections of nicotine (Brown and Kolb, 2001) while another reported significant increases in dendritic length and branch number in the NAc shell of adolescent but not adult rats after continuous nicotine administration (Table 1) (McDonald et al., 2007).

#### Prefrontal cortex

The PFC is a key brain region regulating executive cognitive function, attention, and working memory. Dysregulation of normal signaling in the PFC, paralleled by deficits in cognitive performance, have been observed in disorders such as Alzheimer's disease, schizophrenia, ADHD, and Parkinson's disease (Picciotto and Zoli, 2002). Interestingly, nicotine has been shown to enhance cognition and attention in people suffering from these disorders (Rezvani and Levin, 2001). The PFC has extensive connections to reward and memory hubs, receiving DA innervations from the VTA and providing glutamatergic efferents to the VTA and NAc (Sesack et al., 1989; Carr and Sesack, 2000). It is now well established that drugs of abuse may take over the normal operations of this system, driving impulsivity and compulsive behaviors characteristic of addiction (Lasseter et al., 2010). Recent neuroimaging studies of PFC activity in drug-addicted subjects point to global dysfunction in this region that is associated with a greater incidence of relapse and heavier drug use (Goldstein and Volkow, 2011).

Similar to other brain regions, both α7 and non-α7 nAChR signaling pathways converge in the PFC to modulate both excitatory and inhibitory neurotransmission (see Figure 2). Although glutamatergic pyramidal cells in layer V lack nAChRs, nicotine increases EPSCs and Glutamate release from thalamo-cortical afferents that do express them (Gioanni et al., 1999; Lambe et al., 2003; Couey et al., 2007). There are two populations of GABAergic interneurons, fast spiking and non-fast spiking, with only the latter bearing nAChRs (α7 and α4β2^*^) (Gabbott et al., 1997; Kawaguchi and Kondo, 2002). Activation of both α7 and β2-containing nAChRs on glutamatergic nerve endings enhance the release of excitatory amino acids but do so by different mechanisms (Dickinson et al., 2008). α7 nAChRs, predominately found on ryanodine positive terminals, mediate release by calcium-induced calcium release (CICR) which is coupled to the activation of pre-synaptic extracellular signal-regulated kinase (ERK2) and phosphorylation of synapsin-1. Non-α7 nAChRs recruit voltage-gated calcium channels to induce release of [^3^H] D-aspartate (Dickinson et al., 2008). Additionally, β2-containing nAChRs govern glutamatergic neurotransmission and activation of this receptor subtype by nicotine enhances glutamate release onto layer 5 and 6 pyramidal neurons (Gioanni et al., 1999; Lambe et al., 2003).

***nAChR-mediated synaptic plasticity.*** DA has been shown to be a strong modulator of synaptic plasticity in the PFC by fine tuning glutamatergic transmission and facilitating the induction of LTP or LTD (Otani et al., 2003; Matsuda et al., 2006). Systemic and local administration of nicotine will enhance DA overflow in the medial PFC of rodents (Nisell et al., 1996; Marshall et al., 1997) and both β2-containing and α7 nAChRs influence this response (Table 1) (Livingstone et al., 2009). In rat PFC slices, DA acting through D1 and D2 receptors consistently resulted in LTD and required postsynaptic depolarization and Ca^2+^ influx, but was independent of NMDAR activation (Law-Tho et al., 1995; Otani et al., 1998). In contrast, *in vivo* stimulation of the VTA (250 Hz) induced LTP in hippocampo-PFC projections through cooperative actions of D1 and NMDA receptors (Gurden et al., 1999, 2000).

In the PFC, the relative timing of action potentials in pre- and post-synaptic neurons is critically important for determining the direction of synaptic plasticity, either LTP or LTD, and is referred to as spike-timing-dependent plasticity (STDP) (Markram et al., 1997; Bi and Poo, 1998; Couey et al., 2007). When the pre-synaptic spike occurs before the post-synaptic spike in a time-sensitive manner, robust LTP is induced; in contrast, reversing the order of stimulation will result in LTD. Nicotine will increase the threshold for induction of STDP under the same stimulus conditions by reducing dendritic calcium signals that normally occur with action potential propagation. Pyramidal neurons in the PFC lack nAChRs but GABAergic inhibitory control of these cells is modulated by nicotinic receptors (Couey et al., 2007). The ability of nicotine to eliminate the induction of LTP is attributed to activation of nAChRs on GABA interneurons and glutamatergic cells, which both ultimately increase the excitation of GABA interneurons and enhance inhibition of layer V pyramidal neurons. Application of a GABA_*A*_ receptor antagonist or increasing dendritic calcium signals with burst-like, post-synaptic stimulation blocked nicotine's effects and produced STDP similar to that of control conditions. The authors speculate that one way nicotine may improve cognition is by increasing the signal-to-noise ratio during PFC neural processing (Couey et al., 2007).

### Amygdala and hippocampal complex: learning and memory

Nicotinic receptors have long been known to play a significant role in cognition and disruption of normal nAChR function has been demonstrated in diseases such as Alzheimer's and schizophrenia (Paterson and Nordberg, 2000). nAChR agonists, including nicotine, enhance cognition, memory and promote learning by actions in a number of different brain circuits; however, the underlying mechanisms are still not completely understood (Levin et al., 1994; Socci et al., 1995; Levin and Simon, 1998). An important aspect of addiction is the development of context-drug associations and the formation of memories that link drug-predictive cues to the reinforcing properties of the substance. Studies in both animals and humans have highlighted the importance of associated learning of drug intake with both environmental and internal cues in mediating future drug-seeking and relapse (Fuchs et al., 2008).

The hippocampus and amygdala have been implicated in mediating some of the cognitive-enhancing effects of nicotine, supported by findings that show microinfusion of nAChR agonists can enhance memory-related functions, while antagonists impair them (Ohno et al., 1993; Felix and Levin, 1997). For example, direct infusion of nicotine into basolateral nucleus of the amygdala enhanced working memory and facilitated the acquisition and consolidation of short- and long-term memories; on the other hand, infusion of MLA had opposite effects on memory performance (Barros et al., 2005). Microinfusion of DHβE or MLA into basolateral nucleus of rats resulted in working memory deficits in the radial-arm maze, indicating both α7 and α4β2^*^ nAChRs in this area are involved in normal memory function (Addy et al., 2003). In addition, nicotine dose-dependently stimulates the release of norepinephrine in the amygdala and hippocampus by activating nAChRs localized on norepinephrinergic neurons in the brainstem (Fu et al., 1998). Norepinephrine contributes to memory function and the stress response (Liang et al., 1990; Bremner et al., 1996), offering yet another mechanism by which nicotine modulates these processes.

Few studies have reported the effects of ethanol on nAChRs in these areas. One study reported that co-administration of ethanol (i.p.) and nicotine infusions into the CA1 region or basolateral nucleus of the amygdala produced a significant conditioned place preference, while either drug alone did not; furthermore, this response was blocked by microinjection of mecamylamine into the CA1 or basolateral nucleus of the amygdala (Zarrindast et al., 2010). Taken together, nAChRs in the amygdala and hippocampus play a prominent role in not only learning and memory but also reward-related learning.

#### Hippocampus

From an anatomical prospective, the hippocampus is integrally linked to brain circuits involved in addiction, receiving direct dopaminergic input from midbrain neurons and providing extensive efferent connections to the ventral striatum, amygdala, and PFC (Kelley, 2004). Therefore, alterations in structure or function in the hippocampus may be translated by other brain regions that drive maladaptive behaviors associated with addiction. Most studies investigating the involvement of nAChRs in synaptic plasticity have been conducted in the hippocampus.

**Figure 3:**
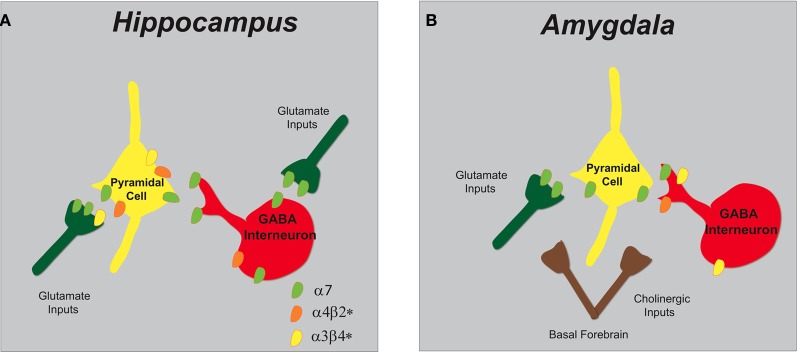
**Schematic representation of nAChR subtypes and circuit function in the hippocampus and amygdala. (A)** In the hippocampus, α7 and α4β2^*^ nAChRs are abundantly expressed on pyramidal cells and inhibitory interneurons. GABAergic interneurons have pre-synaptic α7 nAChRs and somato-dendritic expression of α7 and α4β2^*^ nAChRs. Glutamatergic afferents have predominately pre-synaptic α7 nAChRs and only low levels of α3β4^*^. **(B)** In the amygdala, cholinergic inputs from the basal forebrain synapse in proximity to pre-synaptic nAChRs that modulate both excitatory and inhibitory synaptic transmission. Glutamatergic afferents and pyramidal neurons endow α7 nAChRs and GABAergic interneurons express multiple nAChRs (α7, α4β2^*^, and α3β4^*^).

The α7 and α4β2^*^ nAChRs (see Figure 3) are abundantly expressed on GABAergic interneurons and pyramidal cells within the hippocampus and are capable of modulating intracellular signaling molecules and downstream effectors that govern plasticity (Jones and Yakel, 1997; Vizi and Lendvai, 1999). GABAergic interneuron populations express α7 nAChRs on pre-synaptic terminals, whereas somato-dendritic compartments endow both α7 and α4β2^*^ nicotinic receptors (Radcliffe et al., 1999; Alkondon and Albuquerque, 2001). Furthermore, modulation of glutamate synaptic transmission to pyramidal neurons in the CA1 region is attributed to predominately α7 nAChRs but also to a minimal number of α3β4^*^ nAChRs (Gray et al., 1996; Ji et al., 2001; Alkondon and Albuquerque, 2002).

***nAChR-mediated synaptic plasticity.*** Nicotinic receptors exert a temporally- and spatially-dependent bidirectional control over synaptic plasticity, both *in vitro* and *in vivo* (Table 2). For example, in the CA1 region of hippocampal slices ACh and nicotine can act on post-synaptic receptors of pyramidal neurons to increase intracellular Ca^2+^ which facilitates the conversion of short-term potentiation to LTP by reducing the threshold needed for induction (Fujii et al., 1999; Ji and Dani, 2000; Nakauchi et al., 2007) or by attenuating the inhibitory input of interneurons to pyramidal cells (Ji and Dani, 2000; Yamazaki et al., 2005); these effects are mediated by both the activation of non-α7 nAChRs and the inactivation of α7 nAChRs (Fujii et al., 2000a). Furthermore, blunting the evoked release of inhibitory GABA onto pyramidal cells to facilitate nicotine-induced LTP induction was shown to rely on desensitization of non-α7 nAChRs (Fujii et al., 2000b; Yamazaki et al., 2005; Nakauchi et al., 2007). Additionally, activation of nAChRs on hippocampal interneurons can induce LTP or LTD depending on the exact timing of agonist application in respect to the pre-synaptic stimulation (Ji et al., 2001; Ge and Dani, 2005). Furthermore, activation of α7 nAChRs on pre-synaptic glutamatergic terminals can increase the frequency of miniature EPSCs and enhance glutamate release onto pyramidal neurons offering yet another mechanism for the modulation of plasticity (Gray et al., 1996; Radcliffe and Dani, 1998). In the CA3 region of hippocampal slices, bath application of nicotine can drive the pyramidal cells above threshold in the absence of an action potential by activating pre-synaptic nAChRs located on glutamatergic terminals. Activation of these receptors enhances miniature EPSCs and glutamate release through mobilization of intracellular calcium stores by CICR (Sharma and Vijayaraghavan, 2003).

**Table 2:** **nAChRs modulate synaptic plasticity in the hippocampus and amygdala**.

**Location**	**nAChR subtypes**	**Agonist**	**Outcome**	**Mechanism**	**References**
Hippocampus CA1 region	Post-synaptic activation of non-α7 and inactivation of α7	Nicotine (acute/chronic) and ACh	Reduces threshold for long-term potentiation (LTP) Converts short-term potentiation to LTP	↑ intracellular Ca^2+^ in pyramidal neurons ↑ neuronal excitability	Fujii et al., 1999, 2000a; Ji and Dani, 2000; Nakauchi et al., 2007
	Desensitization of non-α7 on pre-synaptic GABAergic interneurons	Nicotine	Promotes LTP induction	Disinhibition of pyramidal neurons	Ji and Dani, 2000; Fujii et al., 2000b; Yamazaki et al., 2005
	Activation α7 on glutamatergic nerve terminals	Nicotine and ACh	Silent synapses to functional	Facilitation of synaptic transmission	Maggi et al., 2003
	α7 on presynaptic glutamatergic neurons	Nicotine	Promotes LTP induction	Ca^2+^ influx—excitatory post-synaptic currents (EPSCs) ↑ glutamate release onto pyramidal neurons	Gray et al., 1996; Radcliffe and Dani, 1998
	Dependent on type and location of nAChRs	ACh	LTP or LTD induction	Timing of postsynaptic nAChR activation and pre-synaptic stimulation	Ji et al., 2001; Maggi et al., 2004; Ge and Dani, 2005
Hippocampus CA3 region	nAChRs activation on glutamatergic neurons	Nicotine	Brings post-synaptic pyramidal neurons to action potential threshold	Ca^2+^ influx-EPSCs Ca^2+^ induced Ca^2+^ release ↑ glutamate release onto pyramidal neurons	Sharma and Vijayaraghavan, 2003
Dentate gyrus	α7	Nicotine	Enhance tetanus-induced LTP	Depedent on NMDAR and voltage-activated Ca^2+^ channels ryanodine-sensitive Ca^2+^ stores Ca^2+^ induced Ca^2+^ release	Welsby et al., 2006, 2009
	α7 and α4β2*	Nicotine, epibatidine, choline	LTP induction	Requires dopamine input	Matsuyama et al., 2000; Matsuyama and Matsumoto, 2003; Tang and Dani, 2009
Amygdala	α7 and β2-containing	Nicotine *(in vivo* exposure)	Facilitate LTP	NMDAR-dependent Reduces inhibition of pyramidal neurons	Huang et al., 2008

Within the dentate gyrus, induction of LTP by nicotine required activation of mGLuR5 and L-type Ca^2+^ channels, as well as Ca^2+^ release from ryanodine-sensitive stores and was α7 nAChR-dependent (Welsby et al., 2006, 2009). *in vivo* studies in mice showed nicotine or epibatidine, an α4β2 nAChR agonist, dose-dependently induced synaptic plasticity in the dentate gyrus and importantly, required intact midbrain DA signaling (Matsuyama et al., 2000; Matsuyama and Matsumoto, 2003; Tang and Dani, 2009). In the developing brain, long-lasting changes in synaptic transmission were observed following a single exposure to nicotine in the hippocampus. nAChR signaling facilitated the conversion of pre-synaptic silent synapses into functional ones and was shown to be dependent on α7 nAChRs most likely localized on pre-synaptic glutamatergic nerve endings (Maggi et al., 2003). Together these findings strongly imply that the timing and location of nAChR activity are important determinants for synaptic plasticity in the hippocampus.

#### Amygdala

The amygdala is another essential brain region implicated in memory processing, particularly for encoding the emotional and motivational significance of environmental stimuli as well as initiating innate unconditioned responses to aversive situations. It is a central region for integrating sensory and cognitive information through its extensive connections to other limbic structures, the cortex, hippocampus, and thalamus (LeDoux, 1996). In addition, experimental evidence strongly suggests drugs of abuse act on this system and can modify synaptic events, especially during periods of withdrawal (McCool et al., 2010).

Reciprocal connections between the amygdala, hypothalamus and parabrachial nucleus are known to regulate the hypothalamic-pituitary-adrenal axis and autonomic responses to conditioned fear (Takeuchi et al., 1982; Gray et al., 1989). The amygdala also participates in stress- and reward-related behaviors through its connections to the PFC and NAc, respectively (Simpson et al., 2001; Myers-Schulz and Koenigs, 2012; Stuber et al., 2011). The basolateral nucleus of the amygdala is densely innervated by cholinergic projections arising from the basal forebrain (Sah et al., 2003), with cholinergic inputs synapsing on pyramidal neurons (89% of efferents) and GABAergic interneurons (Muller et al., 2011). Different pathways reside within the amygdala and are responsible for various functions regarding the acquisition, expression, and retrieval of fear memories as well as unconditioned behaviors (LeDoux, 2003).

Functional nAChRs expressed on pyramidal cells (somato-dendritic α7), GABAergic interneurons (α7, α4β2^*^, and α3β4^*^), and glutamatergic afferents (α7) modulate synaptic transmission in the amygdala (see Figure 3) (Hill et al., 1993; Perry et al., 2002; Klein and Yakel, 2006). Whole cell patch-clamp recordings demonstrated nicotine increases the frequency of both glutamatergic and GABAergic spontaneous post synaptic currents (PSCs) and this effect was sensitive to the α7-selective antagonist α-bungarotoxin, thus implicating a role of pre-synaptic α7 nAChRs (Barazangi and Role, 2001). Another report showed that activation of predominately α3β4^*^ nAChRs on GABAergic interneurons in the basolateral nucleus of the amygdala were responsible for the enhanced frequency of inhibitory PSCs (Zhu et al., 2005).

***nAChR-mediated synaptic plasticity.*** In the amygdala (Table 2), nicotine has been shown to facilitate LTP in a pathway-specific manner. Robust LTP in amygdala slices from mice that received nicotine treatment for 7 days compared to controls and persisted 72 h after nicotine cessation. Even just one day of nicotine exposure significantly enhanced LTP. The nicotine-induced facilitation of LTP was found to be dependent on both α7 and β2-containing nAChRs and at least partially, to activation of nAChRs on GABAergic interneurons that ultimately reduce inhibition of pyramidal neurons. Furthermore, LTP was completely blocked by D-APV, an NMDAR antagonist, demonstrating the essential role of NMDARs in nicotine-mediated plasticity in the amygdala. The authors suggest that as seen in other brain regions, nicotine may also be acting on pre-synaptic nAChRs on glutamatergic terminals to enhance glutamate release or increasing postsynaptic Ca^2+^ influx through voltage-dependent calcium channels (Huang et al., 2008). At this time, little is known about nicotinic receptor-mediated plasticity in the amygdala. Ethanol is capable of modulating synaptic changes in this circuit but it has yet to be elucidated if and how nicotinic receptors are involved.

## Changes in nAChRs number and function

### Nicotine

A primary mechanism underlying long-lasting synaptic plasticity is a change in the number or expression of membrane-bound receptors. Long-term exposure to nicotine induces an up-regulation of specific subtypes of nAChRs and increases the number of high-affinity nicotinic binding sites across multiple brain regions in the brains of postmortem human smokers (Perry et al., 1999) and nicotine-treated rodents (Schwartz and Kellar, 1983; Flores et al., 1992; Marks et al., 1992; Gentry and Lukas, 2002). The concept of up-regulation of nAChRs is somewhat unexpected and contradictory to what the homeostatic model would predict. Following chronic drug use, receptors are usually down regulated in response to excessive stimulation as an adaptive mechanism to adjust the neural network to a pre-exposure point. Evidence suggests that nicotine causes a rapid desensitization of nAChRs, and this loss in receptor function would promote up-regulation to compensate for the diminished signaling of inactivated receptors over prolonged periods of time (Fenster et al., 1999a,b). These changes result in higher sensitivity to nicotine and have been correlated with nicotine addiction [see review, Govind et al. (2009)].

Several mechanisms have been proposed for nicotine-induced up-regulation of nAChRs and it is quite likely that more than one mechanism is responsible for this phenomenon. There is controversy surrounding how this up-regulation of surface receptors occurs but it does not appear to be due to a change in subunit mRNA transcript levels (Marks et al., 1992; Bencherif et al., 1995; Ke et al., 1998) but has been proposed to be caused by increased translation or alterations in receptor assembly (Wang et al., 1998; Nashmi et al., 2003), trafficking (Harkness and Millar, 2002), and/or decreased receptor turnover (Wang et al., 1998). For example, nicotine has been shown to inhibit the turnover of cell-surface receptors of the α4β2^*^ conformation. The authors, using cell line M10, demonstate that up-regulation of α4β2^*^ nAChRs is due to an intrinsic property of these proteins and results from a conformational change of the receptors that makes their degradation and removal from the cell surface slower (Peng et al., 1994). Another possible mechanism is an increase in receptor trafficking to the cell surface upon long exposures to nicotine (Harkness and Millar, 2002). An increase in the intracellular receptor pool caused by enhanced receptor assembly and/or maturation of the subunits in the endoplasmic reticulum has also been proposed (Nashmi et al., 2003). Additionally, nicotine can reportedly facilitate receptor maturation by acting as a chaperone in the endoplasmic reticulum (Nashmi et al., 2003; Srinivasan et al., 2011). However, membrane-impermanent ligands can also induce up-regulation of surface receptors; therefore, second messengers must exist that are sufficient to drive this response (Whiteaker et al., 1998; Darsow et al., 2005). In order for nicotine-induced up-regulation to occur, nAChRs must pass through the secretory pathway before being inserted into the membrane (Darsow et al., 2005) suggesting that up-regulation is not due to stabilization of nAChRs in the plasma membrane.

The up-regulation of nAChRs varies with subunit composition, cell type and brain region. Amongst the different subtypes of nAChRs, studies have shown the α4β2^*^, α3β2^*^, and α6β2^*^ nAChRs can be activated, desensitized and up-regulated by nicotine concentrations (peak levels between 100 and 500 nM) achieved following cigarette smoking in humans (Nguyen et al., 2003; Parker et al., 2004). The α7 and β4-containing nAChRs appear to be less sensitive to nicotine and require higher concentrations (<10 μM) to activate them. The two accessory subunits α5 and β3 appear to inhibit nicotine-induced receptor up-regulation or down-regulation. In this respect, no up-regulation was reported when α5 subunits were associated with α4β2 nAChRs (Mao et al., 2008) and in the striatum, α6-containing receptors without β3 were down-regulated by nicotine while those containing β3 were unaffected (Perry et al., 2007).

### Ethanol

There are a limited number of studies that have investigated ethanol-induced changes in expression of nAChRs and therefore it is certainly an area of research which should be expounded upon. *In vitro* experiments demonstrated nAChRs are directly affected by ethanol and after long-term exposure these receptors may undergo anatomical and functional changes, possibly by altering receptor expression or composition (Dohrman and Reiter, 2003). In M10 cells, ethanol modulates the number of nAChRs by initially blunting the expression during short exposure (6–72 h) but increasing it with longer incubation periods (96 h). Similar results were found with co-application of relatively high concentrations of nicotine (1 μM) and ethanol (100 mM); moreover, the elevated receptor expression after chronic exposure (96 h) remained up to 7 days following the removal of the drugs (Dohrman and Reiter, 2003). In a different study, long-term consumption of ethanol (5 months) by rats increased the levels of [^3^H]-nicotinic binding in the hypothalamus and thalamus, and decreased the levels in the hippocampus (Yoshida et al., 1982). In ethanol-treated (6 months) mice, small changes in [^3^H]-nicotinic binding were found only in the thalamus and in just one of the mice strains tested, leading the authors to conclude this effect is brain region specific and genetic factors may influence this response (Booker and Collins, 1997). These effects were not seen in mouse brains following short-term (1–2 weeks) ethanol treatment (Burch et al., 1988; de Fiebre and Collins, 1993).

Finally, receptor up-regulation should enhance neuronal excitability and favor induction of drug-induced LTP. Thus, it can be hypothesized that drug exposure leads to a chain reaction of interrelated events: up-regulation of nAChRs—LTP/LTD—enhanced neurotransmitter release—behavioral modifications, which will all contribute to uncontrollable drug use (Vezina, 2004).

## Behavioral implications of plasticity

One behavioral correlate of synaptic plasticity is the manifestation of locomotor sensitization, which is defined as an enhanced locomotor response after repeated exposures to a drug compared to the activity measured during the first drug administration. Increased locomotor response to prolonged nicotine, ethanol, cocaine, amphetamine, and methamphetamine has been extensively studied in rodent animal models and is thought to have relevance to drug seeking and relapse in humans (Steketee and Kalivas, 2011).

Data suggests repeated administration of a drug causes altered dopaminergic and glutamatergic transmission in the mesocorticolimbic system (Vanderschuren and Kalivas, 2000; Pascual et al., 2009) and is associated with the neuroplastic changes discussed thus far in this review. Up-regulation of receptors may not be the sole cause of drug-induced locomotor sensitization, since the timing of these events don't necessarily correlate, but likely plays a role in the development of this behavioral response (Vezina, 2007). nAChRs are up-regulated in the entire brain after long exposures to nicotine but nAChRs in the VTA and NAc are the most probable regions implicated for the induction of sensitization (Parker et al., 2004). Long-lasting behavioral sensitization has been shown to correlate well with LTP, reflecting persistent adaptations in neural mechanisms such as the modulation of synaptic strengths, change in neurotransmitter release, alterations in gene expression and formation of new connections between synapses. In the next section, we will focus on nicotine and ethanol's effect on behavioral sensitization.

### Nicotine and ethanol stimulated locomotor sensitization

Several studies have shown nicotine induces locomotor sensitization in mice and rats by a range of nicotine doses (0.1–2 mg/kg, i.p) and by different schedules of administration (Domino, 2001; Collins and Izenwasser, 2004; Saito et al., 2005). Typically, the first nicotine injection produces locomotor depression which is rapidly overcome by subsequent nicotine exposure and is associated with the development of tolerance to the drug's acute depressant effect (Morrison and Stephenson, 1972). This enhanced locomotor activity in response to repeated nicotine administration is long-lasting (Miller et al., 2001) and central nicotinic receptors play a key role. In support, mecamylamine (Bevins and Besheer, 2001), lobeline (non-selective nAChR antagonists) (Miller et al., 2003), and SSR591813 (α4β2 partial agonist) (Cohen et al., 2003) all blocked induction of sensitization by nicotine. In a separate study, pre-treatment with mecamylamine but not α-bungarotoxin (α7 nAChR antagonist) prevented sensitization, implicating non-α7 nAChRs in mediating the locomotor-stimulant effects of nicotine (Kempsill and Pratt, 2000).

While nicotine–induced sensitization has been widely studied, motor stimulant effects of ethanol have generally received less attention. The development of sensitization to ethanol is predominantly shown in mice. Similar to nicotine-induced locomotor activity, mice were pre-treated with ethanol injections (1.5–2.5 g/kg, i.p) for 7–10 days. Following this exposure, they were challenged with a single injection of ethanol after a period of withdrawal (7–30 days). Results indicated the mice were significantly more sensitive to the locomotor stimulating effects of ethanol during this challenge session and this effect lasted up to 29 days following termination of ethanol administration (Lessov and Phillips, 1998; Itzhak and Martin, 2000; Fish et al., 2002). Under similar circumstances, stimulation of locomotor activity by ethanol consuming rats has also been reported (Hoshaw and Lewis, 2001).

There is a substantial amount of evidence supporting the idea that activation of the DAergic system is required for the emergence of the sensitized locomotor response, with induction of sensitization attributed to the VTA and the expression to the NAc (Mao and McGehee, 2010). Through actions on nAChRs in this system, both nicotine and ethanol influence neuronal activity firing rate (Mereu et al., 1987), bursting activity (Zhang and Sulzer, 2004), and corresponding neurotransmitter release—including DA, GABA, and Glutamate, which are surely contributing to the drugs' locomotor-stimulating effects (Nestby et al., 1997; Guo et al., 1998; Tzschentke, 2001; Lambe et al., 2003; Broadbent et al., 2005; Meyer et al., 2008; Mao et al., 2011). For example, intracranial injections of nicotine directly into the VTA results in locomotor sensitization (Reavill and Stolerman, 1990; Kita et al., 1992) and is associated with an increase in DA and c-Fos-like immunoreactivity (an indicator of neuronal activation) in the NAc (Panagis et al., 1996; Shim et al., 2001). For these reasons, behavioral sensitization induced by nicotine and ethanol can be partially attributed to their actions on nAChRs in the midbrain reward pathway.

### Cross sensitization

While repeated exposure to a single drug can produce behavioral sensitization, sometimes cross-sensitization between drugs is observed. In this type of experiment, animals are repetitively treated with a particular drug for a period of time and then challenged with a different drug after a defined drug-free period. Although the animal has experienced a different drug, locomotor sensitivity to the challenge drug is observed, indicating a common molecular substrate. For example, caffeine, cocaine, and amphetamine have all been shown to produce cross-sensitization to nicotine-induced hyperlocomotion (Collins and Izenwasser, 2004; Celik et al., 2006; Santos et al., 2009). Others studies have demonstrated cocaine and ethanol exhibit cross-sensitization of locomotor effects (Itzhak and Martin, 1999). The findings for nicotine and ethanol are mixed, with some studies reporting no cross-sensitization (Watson and Little, 1999; Darbra et al., 2004) while others report observing this phenomenon (Biala and Weglinska, 2004; Biala and Budzynska, 2010). There are, however, other behavioral measures that clearly illustrate a common molecular interaction between these two substances. In this respect, rats with prior exposure to nicotine show increased ethanol consumption (Blomqvist et al., 1996); furthermore, ethanol-induced locomotor activity and accumbal DA release is blocked by mecamylamine, indicating a pivotal role of nAChRs in ethanol's behavioral effects (Blomqvist et al., 1992; Larsson et al., 2002). In addition, drugs acting through nAChRs, including a partial agonist (varenicline) and non-selective antagonist (MEC), reduce ethanol consumption in both rodents and humans (Le et al., 2000; Steensland et al., 2007; McKee et al., 2009).

## Conclusions

This review has summarized multiple different mechanisms that underlie persistent, long-lasting changes in synaptic efficacy following administration of addictive drugs. It is becoming more and more evident that nicotinic receptors significantly facilitate the induction and maintenance of plasticity—including LTP, LTD, and structural changes—in the hippocampus, amygdala, and mesolimbic dopaminergic system, thus contributing to the molecular underpinnings of nicotine and alcohol addiction. Nicotine exerts its powerful effects by a dynamic, parallel activation, and desensitization of nAChRs. Up-regulation of nAChRs following nicotine treatment reflects a compensatory response to excessive receptor stimulation, and there is compelling experimental evidence to suggest this plays a major part in nicotine dependence. Although few studies have addressed ethanol-induced synaptic plasticity via interactions with nicotinic receptors, ethanol undoubtedly potentiates nAChR currents and drugs targeting nAChRs can attenuate voluntary alcohol consumption in both rodents and humans. Importantly, there is a need to understand the molecular and cellular ramifications of co-administration of nicotine and ethanol due to the high comorbidity of these substances in human addicts. Future studies should aim to unravel the common neural mechanisms shared by these two drugs. This review has touched upon the behavioral outcomes of repeated administration of drugs of abuse, thus suggesting that long-lasting changes in synaptic strength and modification of neurotransmitter release contribute to locomotor sensitization. Both nicotine and ethanol alone clearly induce behavioral sensitization, and cross-sensitization may or may not occur between these two substances. At this time, a large body of literature exists regarding the mechanism of action of nicotine but there is still much to be elucidated pertaining to ethanol's actions at nAChRs for synaptic plasticity and behavioral sensitization.

Clearly, nicotine can enhance cognitive function and propagate LTP and thereby these processes are likely, at least in part, what underlie the highly addictive nature of this compound. Reports from human users of cognitive deficits and strong cue-induced cravings during nicotine withdrawal undoubtedly contribute to the high incidence of relapse. Medications that target neural substrates directly involved in both learning and addiction may offer a novel pharmacotherapeutic approach for nicotine dependence as well as other drugs of abuse. More intriguing yet is the possibility that novel therapeutic avenues may be directed to diminish drug-associated memories or facilitate the formation of new memories with less maladaptive behavioral consequences.

### Conflict of interest statement

The authors declare that the research was conducted in the absence of any commercial or financial relationships that could be construed as a potential conflict of interest.
